# Molecular Insights into Salinity Responsiveness in Contrasting Genotypes of Rice at the Seedling Stage

**DOI:** 10.3390/ijms23031624

**Published:** 2022-01-30

**Authors:** Jingjing Zhang, Tingting Xu, Yiran Liu, Tong Chen, Qiuxin Zhang, Weiyan Li, Hongkai Zhou, Yuexiong Zhang, Zemin Zhang

**Affiliations:** 1 State Key Laboratory for Conservation and Utilization of Subtropical Agro-Bioresources, Guangdong Provincial Key Laboratory of Plant Molecular Breeding, South China Agricultural University, Guangzhou 510642, China; 20191015012@stu.scau.edu.cn (J.Z.); 20193137033@stu.scau.edu.cn (T.X.); yiranliu@stu.scau.edu.cn (Y.L.); chentong5152@stu.scau.edu.cn (T.C.); zhangqiuxin@stu.scau.edu.cn (Q.Z.); 20211015008@stu.scau.edu.cn (W.L.); 2 College of Agriculture, Guangdong Ocean University, Zhanjiang 524088, China; zhouhk@gdou.edu.cn; 3 Guangxi Key Laboratory of Rice Genetics and Breeding, Rice Research Institute, Guangxi Academy of Agricultural Sciences, Nanning 530007, China

**Keywords:** rice, salinity response, HAKs, seedling stage, transcriptome, AsA–GSH pathway

## Abstract

Salinity is one of the most common unfavorable environmental conditions that limits plant growth and development, ultimately reducing crop productivity. To investigate the underlying molecular mechanism involved in the salinity response in rice, we initially screened 238 rice cultivars after salt treatment at the seedling stage and identified two highly salt-tolerant cultivars determined by the relative damage rate parameter. The majority of cultivars (94.1%) were ranked as salt-sensitive and highly salt-sensitive. Transcriptome profiling was completed in highly salt-tolerant, moderately salt-tolerant, and salt-sensitive under water and salinity treatments at the seedling stage. Principal component analysis displayed a clear distinction among the three cultivars under control and salinity stress conditions. Several starch and sucrose metabolism-related genes were induced after salt treatment in all genotypes at the seedling stage. The results from the present study enable the identification of the ascorbate glutathione pathway, potentially participating in the process of plant response to salinity in the early growth stage. Our findings also highlight the significance of high-affinity K^+^ uptake transporters (HAKs) and high-affinity K^+^ transporters (HKTs) during salt stress responses in rice seedlings. Collectively, the cultivar-specific stress-responsive genes and pathways identified in the present study act as a useful resource for researchers interested in plant responses to salinity at the seedling stage.

## 1. Introduction

Rice is one of the most important cereal grains and serves as a staple food for more than half of the world’s population. Soil salinity significantly adversely affects plant metabolism through osmotic stress and ion toxicity, affecting plant growth and development, as well as reducing water uptake. Salinity is considered to be one of biggest environmental constraints to rice production, leading to substantial losses worldwide, especially in arid and semi-arid regions [1]. Over the past decades, emerging evidence has revealed the physiological, biochemical, and molecular mechanisms of salt tolerance in different plant species, including *Arabidopsis* and rice [2,3,4]. Currently, breeding salinity-resistant cultivars is the most promising solution for salinity tolerance in high-yielding crops; therefore, a better understanding of the mechanism of plant response to salt stress and the mining of salt tolerance-associated genetic resources is crucial for salt-tolerant crop cultivation.

In general, plant growth responds to salinity in two phases: a rapid response to an increase in osmotic pressure, followed by a slower response after the accumulation of Na^+^ in mature tissues, leading to ion toxicity affecting plant growth and development [5]. Osmotic stress causes a multitude of physiological changes, including cell membrane distortion, protein aggregation, DNA damage, disordered reactive oxygen species (ROS) production, severe ion imbalance, and decreased photosynthetic activity [6]. Plants as sessile organisms have evolved various physiological and biochemical strategies to protect them from salt stress, for example, plant cells maintain ion homeostasis by ion uptake and compartmentalization, and Na^+^ enters the cytoplasm and is subsequently transported to the vacuole by an Na^+^/H^+^ antiporter, which is extensively involved in a Salt Overly Sensitive (SOS) stress signaling pathway [7,8,9]. The prominent genes working in this pathway consist of SOS1, a plasma membrane Na^+^/H^+^ antiporter; SOS2, a serine/threonine protein kinase; and SOS3, encoding a myristoylated calcium-binding protein that functions as Ca^2+^ sensors [6,10]. Therefore, activation of the SOS signaling pathway is considered a crucial mechanism for Na^+^ exclusion and ion homeostasis in response to salinity stress in plants.

As Na^+^ and K^+^ homeostasis regulation is key to the survival of plants under saline conditions, the uptake of Na^+^ at the root–soil boundary occurs mainly through nonselective cation channels, such as cyclic nucleotide-gated channels (CNGCs), high-affinity K^+^ transporters (HKTs), and high-affinity K^+^ uptake transporters (HAKs) [11]. HKTs belong to an important class of integral membrane proteins that facilitate cation transport across the plasma membranes of plant cells [12]. It is generally recognized that the HKT-mediated transport of Na^+^ is a crucial component of salinity tolerance in different plant species, such as wheat [13,14,15], *Arabidopsis* [16,17,18,19], and rice [20,21,22]. In rice, the *HKT* family consists of nine genes that can be divided into two subfamilies based on amino acid sequence similarity and differences in Na^+^ and K^+^ transport capacity [23,24]. The most well-studied HKT in rice is probably *OsHKT2;1*, which is an unusual class II transporter as it contains a serine residue in the first pore domain, whereas its Na^+^ transport capacity is similar to that of class I members [25]. Rice *hkt2;1* transposon insertion mutant displayed significantly reduced growth in comparison to those of wild-type plants under low-Na^+^ and K^+^ starvation conditions. Notably, *hkt2;1* accumulated less Na^+^, but not less K^+^, and further evidence revealed that *OsHKT2;1* mediates a large Na^+^ influx component into K^+^-starved roots for growth [26]. Although the involvement of some HKT transporters in Na^+^ and K^+^ is well established, the molecular mechanism of the transport is unclear.

In the present study, highly salt-tolerant, moderately salt-tolerant, and salt-sensitive rice genotypes with different salinity responses were identified from 238 rice cultivars after salinity treatment at the seedling stage. We further provided a global transcriptome analysis of three genotypes in water and salinity treatments. Several metabolic pathways, including starch and sucrose metabolism, phenylpropanoid biosynthesis, and glutathione metabolism pathways, were altered after salinity treatment in both highly salt-tolerant and salt-sensitive plants. The results also highlight the significance of the HAKs, HKTs, and ascorbate–glutathione (AsA–GSH) pathway during salt stress responses. Taken together, the present study provides a better understanding of the molecular mechanisms of plant responses to salinity stress at the seedling stage.

## 2. Results

### 2.1. Phenotypic Characterization of Resistant and Sensitive Rice Cultivars in Response to Salinity Stress at Seedling Stage

A total of 238 rice cultivar seedling genotypes were evaluated for their response to salt stress at the seedling stage. Seeds from different rice cultivars were germinated in distilled water and 150 mM NaCl solution, and the seedling percentage (SP) in water and salt solutions was recorded at 7 days after germination (DAG). The relative damage rate (RDR) was calculated to evaluate salt tolerance and compile salt tolerance rankings. Genotypes were divided into five groups based on the RDR parameter, including highly salt-tolerant (RDR ≤ 20%), salt-tolerant (20% < RDR ≤ 40%), moderately salt-tolerant (40% < RDR ≤ 60%), salt-sensitive (60% < RDR ≤ 80%), and highly salt-sensitive (80% < RDR ≤ 100%). Only two cultivars were ranked as highly salt-tolerant, including SanXiang628 (SX628) and IAPAR9 (Table S1), and five cultivars were recognized as salt-tolerant (Table S1). The vast majority of cultivars displayed salt sensitivity, with 94.1% of cultivars ranked as sensitive and highly salt- sensitive (Figure S1, Table S1).

**Figure 1:**
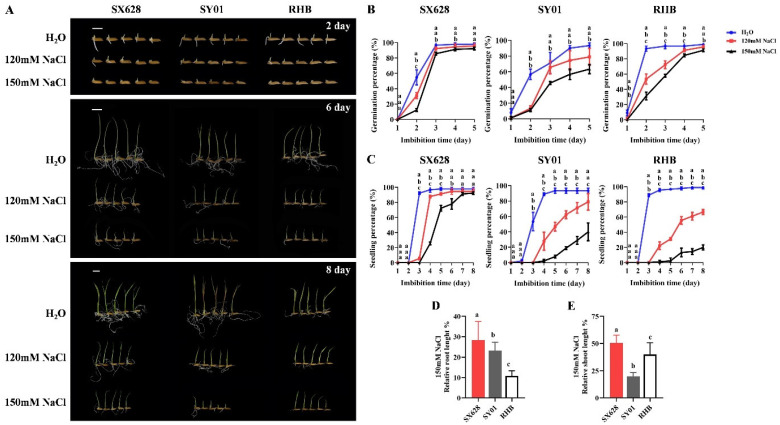
Comparison of seed germination and seedling establishment between SanXiang628 (SX628, highly salt-tolerant), SY01 (moderately salt-tolerant), and RHB (salt-sensitive) under control conditions and salinity stress. (**A**) Seed germination and seedling phenotypes in SX628, SY01, and RHB under control condition, and 120 mM and 150 mM NaCl solutions. Bars = 10 mm. (**B**,**C**) Statistical analysis of germination percentage (**B**) and seedling percentage (**C**) in SX628, SY01, and RHB under control condition, and 120 mM and 150 mM NaCl solutions after germination. Values are mean ± SD (*n* = 30). Data were analyzed using two-way ANOVA with post hoc Tukey tests (letters indicate significant differences between samples at *p* < 0.05). (**D**,**E**) Relative root length (**D**) and relative shoot length (**E**) at 8 DAG in SX628, SY01, and RHB under control condition and 150 mM NaCl solution. Data were analyzed using two-way ANOVA with post hoc Tukey tests (letters indicate significant differences between samples at *p* < 0.05).

Next, we selected highly salt-tolerant (SX628), moderately salt-tolerant (SY01), and salt-sensitive (RHB) for further analysis. Three rice genotypes with different salinity responses were treated with distilled water, and 120 mM and 150 mM NaCl solutions. All genotypes displayed a significantly decreased germination percentage at 2 DAG in 120 mM and 150 mM NaCl compared to that of the control (Figure 1A,B). Notably, the final germination percentages of SY01 and RHB significantly decreased under the 150 mM NaCl treatment at 5 DAG (Figure 1B). We further calculated the seedling percentage in different plants under salt treatment, and rice seedlings were considered established when the root length reached the seed length and the shoot length reached half of the seed length. As depicted in Figure 1C, in SX628 (highly salt-tolerant), although the SP in 120 mM and 150 mM NaCl was lower than that of the control between 3 and 5 DAG, no obvious final SP difference was detected between the control and salinity treatments (Figure 1C). Significantly decreased SPs were detected in both 120 mM and 150 mM NaCl compared to that in water at 3 DAG in SY01 (moderately salt-tolerant) and RHB (salt-sensitive), and the final SPs in SY01 and RHB were 39.9% and 20% under the 150 mM NaCl treatment, respectively (Figure 1C). Relative shoot and root lengths of SX628, SY01, and RHB were measured in the control and 150 mM NaCl, and root growth was inhibited in RHB because the relative root length was significantly shorter than those in SX628 and SY01 (Figure 1A,D), whereas the relative shoot length in RHB was significantly longer than that in SY01 (Figure 1A,E), indicating that salinity mainly inhibits root growth in RHB at the seedling stage.

### 2.2. Transcriptome Analysis of Response to Salinity in Contrasting Genotypes Revealed That Several Metabolic Pathways Were Altered in Highly Salt-Tolerant and Salt-Sensitive Plants

**Figure 2:**
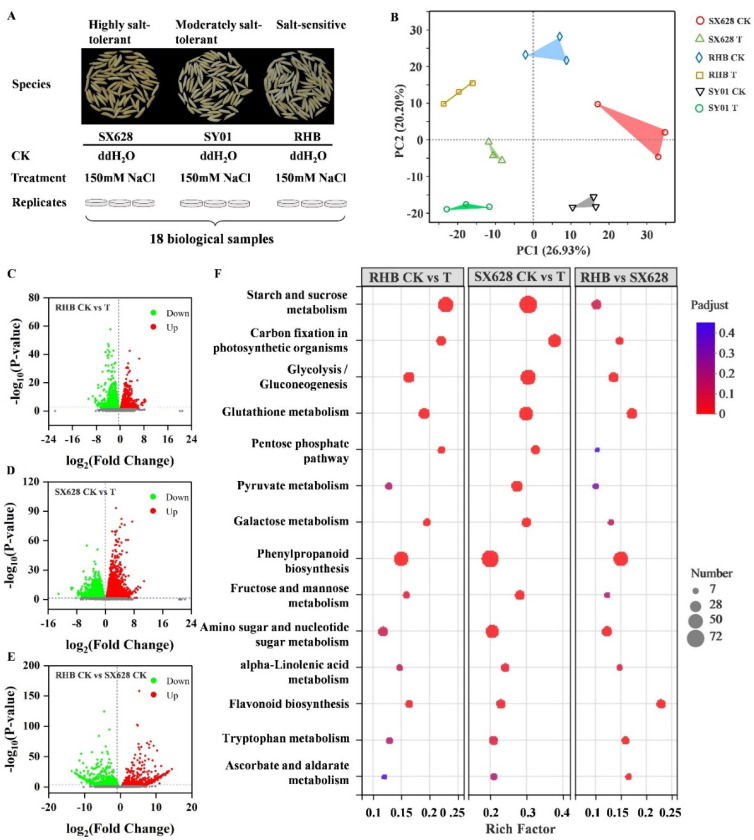
Experimental design and analysis of upregulated and downregulated genes after salt treatment in different rice genotypes at seedling stage. (**A**) Experimental design. Highly salt-tolerant (SX628), moderately salt-tolerant (SY01), and salt-sensitive (RHB) seeds were treated with water and 150 mM NaCl solution. RNA-seq data were collected from three independent biological replicates of each sample, and a total of 18 libraries were constructed. (**B**) Principal component analysis (PCA) of transcriptomes of all 18 RNAseq libraries. (**C**) Volcano plot of DEGs (1095 upregulated and 1551 downregulated genes) in RHB (salt-sensitive) between control and salinity treatment. (**D**) Volcano plot of DEGs (2039 upregulated and 2554 downregulated genes) in SX628 (highly salt-tolerant) between control and salinity treatment. (**E**) Volcano plot of DEGs (1226 upregulated and 1320 downregulated genes) between RHB and SX628. (**F**) The enrichment of the KEGG pathway of DEGs between different comparisons, where the size of the circle represents the number of genes.

To further explore the underlying molecular mechanism involved in the response to salinity in rice, highly salt-tolerant (SX628), moderately salt-tolerant (SY01), and salt-sensitive (RHB) seeds were treated with the 150 mM NaCl solution during seed germination, with water treatment as a control (Figure 2A). A total of 18 libraries were constructed (Figure 2A), and 42.52–60.29 million clean reads were generated in different samples at the seedling stage (Table S2), representing 137.73 GB of clean reads, and 89.62–92.77% of clean reads could be mapped to the *Indica* 9311 reference genome ASM465v1 (Table S3). A total of 42,031 transcripts were assembled from these transcriptomes, and 32,937 genes were expressed at detectable levels. Different types of transcripts were identified in the transcriptome of different samples (Table S4), and alternative splicing (AS) events were also detected (Table S5), including skipped exon (SE), retained intron (RI), alternative 5′ splice site (A5SS), alternative 3′ splice site (A3SS), and mutually exclusive exons (MXE). Principal component analysis (PCA) displayed a clear distinction between the three cultivars under control and salinity stress conditions (Figure 2B). To explore the expression regulation upon salt treatment, the transcriptomes of highly salt-tolerant (SX628) and salt-sensitive (RHB) cultivars were compared after treatment with NaCl. The results showed that the number of DEGs in highly salt-tolerant plants were larger than those detected in salt-sensitive plants (Figure 2C,D). Specifically, 2646 DEGs were detected in the comparison of salt-sensitive RHB CK vs. RHB T (1095 upregulated and 1551 downregulated genes, Figure 2C), and we further performed KEGG enrichment analyses for the DEGs as KEGG provides an insight into the molecular understanding of the rice response to salinity stress conditions. Notably, KEGG analysis categorized these DEGs mainly related to starch and sucrose metabolism, and phenylpropanoid biosynthesis (Figure 2F). In addition, 4593 DEGs were detected in the comparison of highly salt-tolerant SX628 CK vs. SX628 T (2039 upregulated and 2554 downregulated genes, Figure 2D), and KEGG analysis categorized these DEGs mainly related to metabolic pathways, such as starch and sucrose metabolism, phenylpropanoid biosynthesis, and glutathione metabolism (Figure 2F).

We further compared transcriptome profiles in highly salt-tolerant and salt-sensitive plants to investigate the differential genes’ regulation between two contrasting genotypes, a total of 2546 DEGs (1226 upregulated and 1320 downregulated genes, Figure 2E) were detected in this comparison, and these DEGs are related to several metabolic pathways, including phenylpropanoid biosynthesis, glycolysis, glutathione metabolism, amino sugar and nucleotide sugar metabolism, flavonoid biosynthesis, and tryptophan metabolism (Figure 2F). Collectively, the results suggested that several metabolic pathways were altered after salinity treatment in both highly salt-tolerant and salt-sensitive plants.

### 2.3. Activation of Genes in Rice after Salinity Treatment at Seedling Stage Is Associated with Starch and Sucrose Metabolism Pathways

**Figure 3:**
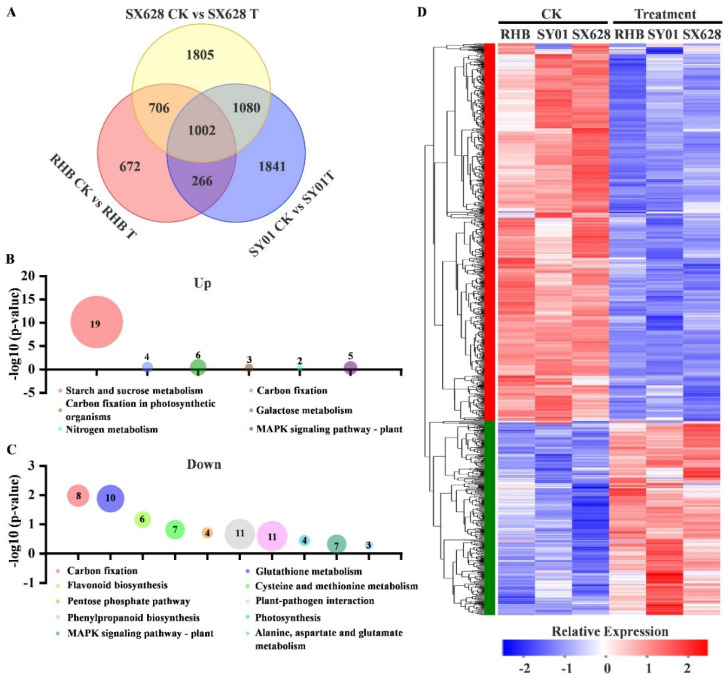
Differentially expressed genes (DEGs) in highly salt-tolerant, moderately salt-tolerant, and salt-sensitive plants under control and salinity stress conditions. (**A**) Venn diagram showing DEGs among highly salt-tolerant, moderately salt-tolerant, and salt-sensitive plants after salinity treatments. (**B**,**C**) Pathway analysis of upregulated (**B**) and downregulated (**C**) DEGs among highly salt-tolerant, moderately salt-tolerant, and salt-sensitive plants after salinity treatments. (**D**) DEGs between plants after salinity treatments. Each row represents a gene, and each column represents a different sample.

To identify significant gene upregulations after salinity treatment at the seedling stage, we investigated overlapping differentially expressed genes (DEGs) that were simultaneously upregulated or downregulated in all genotypes, i.e., highly salt-tolerant, moderately salt-tolerant, and salt-sensitive plants. As a result, 1002 DEGs were identified in three comparisons (SX628 CK vs. SX628 T, SY01 CK vs. SY01 T, RHB CK vs. RHB T); specifically, 340 and 662 DEGs were significantly upregulated and downregulated in these comparisons (Figure 3A,D). KEGG analysis categorized these upregulated DEGs as mainly related to starch and sucrose metabolism pathways (Figure 3B), and the downregulated DEGs were mainly related to several metabolic pathways, such as carbon fixation and glutathione metabolism (Figure 3C).

**Figure 4:**
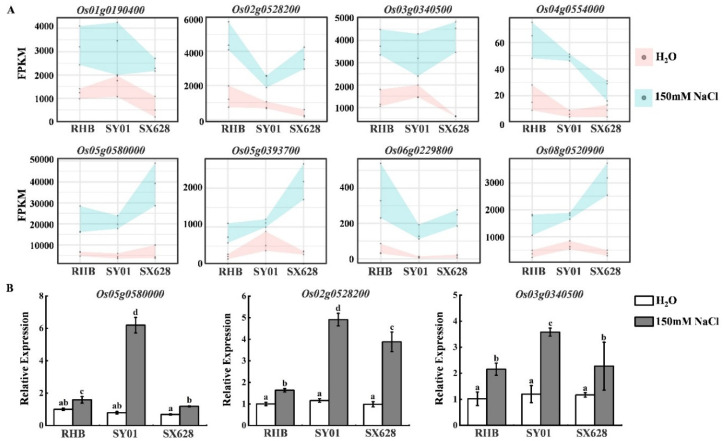
Differentially expressed genes (DEGs) enriched in starch and sucrose metabolism pathways and validated by RT-qPCR. (**A**) Expression of starch and sucrose metabolism-related genes in in three comparisons (SX628 CK vs. SX628 T, SY01 CK vs. SY01 T, RHB CK vs. RHB T) under control and salinity stress conditions. The range of expression values is defined as the minimum and maximum FPKM values collected from three replicates. (**B**) RT-qPCR analysis of three starch and sucrose metabolism-related genes in RHB, SY01, and SX628 under control and salinity stress treatment. Letters indicate significant difference between different samples (*p* < 0.05) as determined using ANOVA.

KEGG enrichment analysis of upregulated DEGs indicated that the starch and sucrose metabolism pathway was enriched, suggesting that this pathway is potentially involved in the response to salinity stress in rice. A total of 19 upregulated genes were identified in the starch and sucrose metabolism pathway (Figure 4A, Table S6). To verify the results of the upregulated genes in the RNA-seq analysis, several potential key genes were chosen to check expression patterns in different samples using RT-qPCR. As depicted in Figure 4B, *OsAGPL1* (*Os05g0580000*), *OsBEIIb* (*Os02g0528200*), and *OsSUS4* (*Os03g0340500*) were induced after salinity treatment, which was similar to the results of the RNA-seq analysis (Figure 4B).

Several hormone-related genes were also detected. Abscisic acid (ABA) biosynthesis gene *OsAAO3* (*Os03g0790900*) was found to be significantly upregulated in both highly salt-tolerant and salt-sensitive plants after treatment (Table S7), whereas the ABA signal-related gene *OsPYL4* (*Os03g0297600*) was significantly downregulated in highly salt-tolerant and salt-sensitive plants after treatment (Table S7, Figure S2). A few ABA signal-related genes showed diverse expression patterns in highly salt-tolerant and salt-sensitive plants, such as *OsPP2C1* (*Os09g0325700*) and *OsSnRK2* (*Os04g0629300*), which were significantly upregulated in highly salt-tolerant plants, whereas their expressions were unchanged in moderately salt-tolerant and salt-sensitive plants after salinity treatment (Table S7, Figure S2).

### 2.4. Dynamic Expression of HAKs and HKTs among Contrasting Rice Cultivars and Salinity Treatment at Seedling Stage

**Figure 5:**
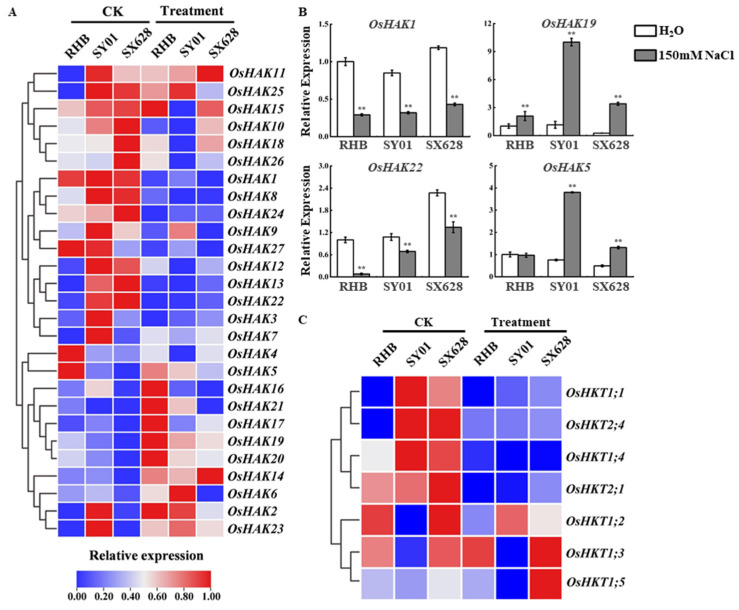
Expression profiling of *OsHAK*s and *OsHKT*s in highly salt-tolerant, moderately salt-tolerant, and salt-sensitive plants under control and salinity treatments. (**A**,**C**) Heatmap of *OsHAK*s (**A**) and *OsHKT*s (**C**) in different samples in control and salinity treatments. (**B**) RT-qPCR analysis *OsHAK*s in RHB, SY01, and SX628 under control and salinity condition. ** *p*-value < 0.01, two-tailed, two-sample Student’s *t*-test.

Based on our previous analysis of DEGs in different comparisons, high-affinity K^+^ uptake transporters (HAKs) and high-affinity K^+^ transporters (HKTs) were suggested to be potentially involved in the response to salinity. This is not surprising, because accumulating evidence has demonstrated that HATs and HKTs play important roles in abiotic stress responses. Thus, we systematically analyzed the abundance of HATs and HKTs in different samples and salinity treatments at the seedling stage. Overall, 27 HAKs have been identified in the rice genome, and seven to nine HKT transporters have been identified in rice, depending on the cultivar [27,28]. *OsHAK1* and *OsHAK19* were significantly downregulated and upregulated after salinity treatment in all cultivars, including highly salt-tolerant, moderately salt-tolerant, and salt-sensitive (Figure 5A); *OsHAK1* showed no obvious difference between the different cultivars (RHB vs. SY01, RHB vs. SX628, SY01 vs. SX628), whereas *OsHAK19* was downregulated in SX628 compared to those in SY01 and RHB (Figure 5A). *OsHAK13* and *OsHAK22* showed similar expression patterns, namely, they were significantly downregulated in SX628 and SY01 after salinity treatment and were upregulated between RHB vs. SY01 and RHB vs. SX628 (Figure 5A). Notably, *OsHAK5* was upregulated in SX628 and SY01 after treatment but was downregulated in different cultivars (RHB vs. SY01, RHB vs. SX628, SY01 vs. SX628, Figure 5A). Several HAKs, such as *OsHAK1*, *OsHAK5*, *OsHAK19*, and *OsHAK22*, were chosen to check expression levels in different cultivars under control and salinity conditions using RT-qPCR, and the expression pattern was similar to the results of RNA-seq analysis (Figure 5B). Interestingly, *OsHKT2;1* was significantly downregulated in all genotypes after salt treatment, suggesting that *OsHKT2;1* is potentially involved in the salinity stress response at the seedling stage (Figure 5C).

### 2.5. Several Genes Related to the AsA–GSH Pathway Displayed Significant Changes under Salinity Treatment in Contrasting Genotypes

**Figure 6:**
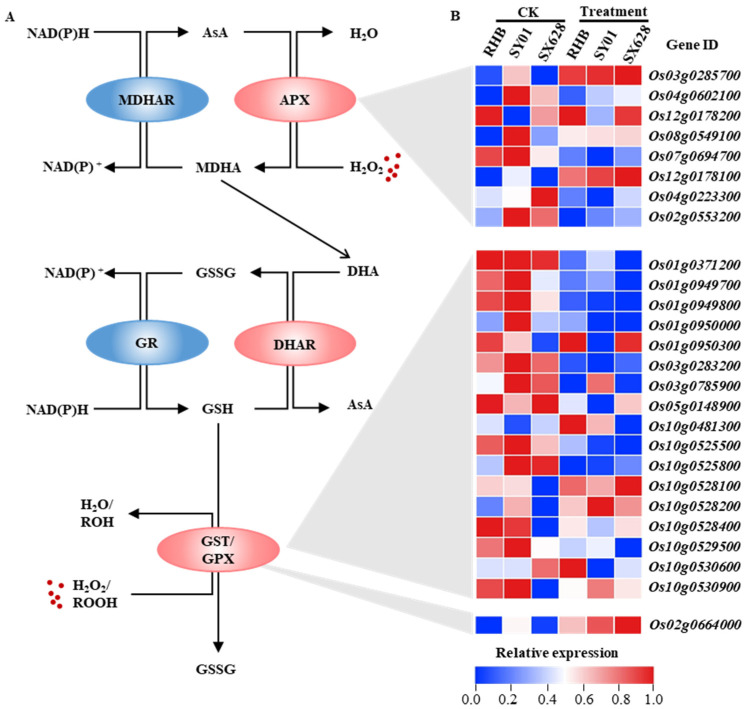
Gene expression in the Ascorbate–Glutathione (AsA–GSH) pathway. (**A**) AsA–GSH pathway in plants. Abbreviations: MDHAR, monodehydroascorbate reductase; APX: ascorbate peroxidase; GR: glutathione reductase; DHAR: cytosolic dehydroascorbate reductase; GST: glutathione S-transferase; GPX: glutathione peroxidase. (**B**) Heatmap of AsA–GSH pathway-related genes in highly salt-tolerant, moderately salt-tolerant, and salt-sensitive plants under control and salinity treatments.

A number of antioxidants and nonenzymatic antioxidants, such as ascorbate (AsA), glutathione (GSH), and ascorbate peroxidase (APX), have been proven to be key players in the response to abiotic stress, and accumulating evidence has revealed the crucial roles of the Ascorbate–Glutathione (AsA–GSH) pathway during this process; thus, we investigated the gene expression levels corresponding to key enzymes of the AsA–GSH pathway after salinity treatment at the seedling stage (Figure 6A). Glutathione *S*-transferase (*GST, Os01g0950300*, *Os10g0528100*, *Os10g0528200*, and *Os10g0530900*) genes generally display significantly decreased or unchanged expression levels in salt-sensitive plants after treatment, whereas several GST genes were induced in highly salt-tolerant plants (Figure 6B). *APX* genes are known to regulate signal flux to maintain H_2_O_2_ homeostasis. Notably, different APXs displayed diverse expression patterns in plants; for example, *OsAPX1* (*Os03g0285700*) and *OsAPX8* (*Os02g0553200*) were found to be significantly upregulated and downregulated in all genotypes after salinity treatment (Figure 6B), suggesting multifaceted roles of *OsAPXs* in response to salt stress conditions. Additionally, the expression levels of several genes related to the AsA–GSH pathway were found to be upregulated or downregulated under salt treatment; for example, *OsGPX3* (*Os02g0664000*) was significantly upregulated in highly salt-tolerant plants after treatment compared to that in the control (Figure 6B).

## 3. Discussion

Salinity is a major and complex abiotic stress that inhibits plant growth and reduces crop yield, and the ability to tolerate salt stress varies widely among different species and cultivars. In the present study, salt tolerance among 238 rice cultivars was evaluated according to the RDR parameter at the seedling stage. The majority of cultivars (94.1%) were ranked as salt-sensitive and highly salt-sensitive (Figure S1, Table S1), and only 0.84% and 2.1% cultivars displayed high salt tolerance and salt-tolerance, respectively, after the 150 mM NaCl treatment at seedling stage. Evaluating plant genotypes at an early growth stage requires less time and resources than assessments at adult stages. Emerging evidence has shown that the underlying molecular mechanism involved in the salinity response of rice at the seedling stage is different from that at vegetative and reproductive stages. Identification of salt tolerant cultivar seedlings could provide information regarding the tolerance mechanisms present at early growth stages.

Gene regulation plays a dominant role in plant responses to biotic and abiotic stresses, and dynamic and intricate transcriptional regulatory networks enable plants to detect external signals. Global transcriptome profiling by RNA-seq has been widely applied to explore the molecular mechanisms underlying the interactions between several crops and saline conditions. For example, Shankar et al., (2016) compared the transcriptomes of a drought-tolerant, salinity-tolerant rice cultivar and a susceptible cultivar under control and stress conditions, and their results revealed that transcripts encoding thioredoxin and those involved in phenylpropanoid metabolism were upregulated in drought-tolerant genotypes, whereas transcripts involved in wax and terpenoid metabolism were upregulated in the salinity-tolerant genotype [29]. Cartagena et al., (2021) investigated the RNA-seq transcriptome profiles of nodal root, L-type lateral roots, and S-type lateral roots in salt-tolerant and salt-sensitive rice varieties and identified several stress-inducible genes including *NAC*, *WRKY*, and *MYB* in the sensitive genotype, whereas genes involved in ion and sugar transport were found to be involved in the regulation of salt tolerance in the roots of salt-tolerant genotypes [30]. Therefore, global transcriptome profiling provides a comprehensive overview of the transcriptional regulation in different samples.

A comparison between two or more genotypes with contrasting salt responsiveness is beneficial for the identification of new tolerance genes in plants. Here, to investigate the molecular mechanisms of salinity tolerance in rice at the seedling stage, transcriptome profiles were collected from highly salt-tolerant (SanXiang628, SX628), moderately salt-tolerant (SY01), and salt-sensitive plants (RHB) seeds treated with the 150 mM NaCl solution during seed germination, with water as a control. We focused on overlapping DEGs induced in all genotypes after salinity treatment and categorized the overlapping upregulated DEGs as mainly related to starch and sucrose metabolism pathways (Figure 3B), suggesting that starch and sucrose potentially participated in response to salinity stress in plants at the seedling stage.

Starch and sucrose are the principal end-products of photosynthesis and are important for energy and carbon skeleton supply [31]. Starch has been suggested to be a determinant of plant fitness during abiotic stress, including high salinity, drought, and extreme temperatures [32]. Starch plays fundamental roles in plant development and growth, because it is consumed to provide a carbon source to plants at night. Additionally, starch is considered a crucial molecule in mediating plant responses to abiotic stress. Some studies have revealed that starch metabolism is involved in salinity stress in different plant species. For example, the levels of starch were substantially decreased after 150 mM salt stress conditions in *Arabidopsis* [33]; starch content also decreased in NaCl-stressed rice seedling leaves during the daytime [34]. However, in some cases, starch levels increased in response to salinity stress, and starch accumulated in the chloroplasts after salt stimulation in *Thellungiella halophile* [35]. Sucrose functions as an osmolyte to prevent tissue damage during water stress [36], and sucrose transport and distribution are important processes to maintain sugar homeostasis during responses to abiotic stresses [37]. Recently, Mathan et al. (2021) showed that the levels of several metabolites, including sucrose, glucose, and fructose, were substantially increased in IR64, which is a drought- and salt-sensitive variety, and further demonstrated that *OsSWEET13* and *OsSWEET15* are major SWEET transporters that regulate sucrose transport and content under abiotic stresses in rice [36].

In the present study, we systematically analyzed HAK and HKT family expression patterns in three cultivars germinated under control and salinity stress at the seedling stage. Several HAK genes displayed significant changes under salt treatment, such as *OsHAK5,* which was upregulated after treatment in SX628 (highly salt-tolerant) and SY01 (moderately salt-tolerant), whereas the expression level of *OsHAK5* was unchanged in RHB (salt-sensitive plant) after salinity treatments (Figure 5A), suggesting that *OsHAK5* potentially participates in the salinity stress response in plants at the seedling stage. *OsHAK5* is localized in the plasma membrane and displays enhanced expression levels after K starvation for 10 days [38]. Additionally, overexpression of *OsHAK5* increased the K^+^ concentration ratio in the shoots and salt stress tolerance, whereas knockout of *OsHAK5* decreased the K^+^ concentration ratio in the shoots, resulting in sensitivity to salt stress in rice [38]. Recent studies have highlighted that *OsHAK5* also mediates root morphology and shoot architecture regulation by changes in extracellular pH, PM polarization, and auxin distribution in rice [39], suggesting that *OsHAK5* has multifaceted roles in various biological processes in plants.

It is generally recognized that reactive oxygen species (ROS) detoxification is an essential component of salinity stress tolerance mechanisms. ROS scavenging is achieved by two efficient antioxidant systems that include enzymatic components, comprising catalase (CAT), superoxide dismutase (SOD), ascorbate peroxidase (APX), and nonenzymatic antioxidants, comprising ascorbic acid (AsA), reduced glutathione (GSH), and flavonoids. Within the antioxidant system, the AsA–GSH pathway plays a central role in regulating ROS homeostasis. The present study revealed that several AsA–GSH pathway-related genes displayed significant changes under salinity treatment, and a series of Glutathione S-transferase (GST) genes were downregulated in response to salt stress conditions (Figure 6), by catalyzing the conjugation of tripeptide glutathione (GSH) to a variety of electrophilic and hydrophobic substrates. It has been well documented that GSTs are involved in regulating oxidative stress metabolism; for example, *AtGSTU17* was reported to be a negative regulator of the stress-mediated signal transduction pathway in response to drought and salt stress [40], and overexpression of *OsGSTU4* resulted in an improved tolerance to salinity and oxidative stress in transgenic *Arabidopsis* plants [41]. The differential expression of AsA–GSH pathway-related genes has been observed in response to salinity stress conditions in contrasting genotypes (Figure 6), suggesting that the AsA–GSH pathway is potentially involved in salinity stress in plants at the seedling stage.

In conclusion, contrasting genotypes with different salinity responses at the seedling stage were ranked from 238 rice cultivars, and the present study provides molecular insight into the underlying mechanisms involved in salinity resistance in plants at the seedling stage. Tolerant genotypes will require confirmation using field studies of the adult stages; thus, further research will be necessary to understand the functions of DEGs at the seedling stage and to unravel the mechanism of resistance to salinity at both seedling and adult stages, with the aim of developing potentially high-productivity rice plants for crop breeding under salinity stress conditions.

## 4. Materials and Methods

### 4.1. Evaluation of Seed Germination

All seeds were harvested at the mature stage and dried at 40 °C for 7 days to break seed dormancy, and 30 seeds of each cultivar were selected and placed in Petri dishes (diameter 9 cm) with 10 mL of distilled water and 150 mM NaCl solution at 30 ± 1 °C in a climate chamber for 8 days under a 12 h light/12 h dark cycle. A total of 238 rice cultivar seedling genotypes were evaluated for their response to salt stress. Seed germination rate, seedling percentage (SP), and relative damaging rate (RDR) were recorded. The parameters used for the evaluation of seed germination were evaluated as previously described [42]. Briefly, rice seedlings were considered established when the root length reached the seed length and the shoot length reached half of the seed length. The SP was calculated at 2, 4, 6, and 8 DAG; SP (%) = (number of final seedlings/total grains) ×100%; RDR (%) = [(SP of CK—SP of salt treatment)]/SP of CK × 100%. Three replications were conducted, and the mean value was used for the data analysis.

### 4.2. Sample Preparation and RNA Sequencing

Eight-day-old seedlings of SX628, SY01, and RHB were divided into two groups and each was treated with either water or 150 mM NaCl solution. The whole plants were harvested, immediately frozen in liquid nitrogen, and stored at −80 °C. Total RNA from each sample was extracted using TRIzol reagent (Takara, Japan) following the manufacturer’s instructions. Before library preparation, RNA concentrations, purities, and integrity numbers were checked using NanoDrop2000 and Agilent 2100 Nano. RNA-seq library construction and sequencing were performed by Mahorbio company (Shanghai, China) using the Illumina Truseq^TM^ RNA sample prep Kit. Library quality was confirmed with fastp software, and clean reads were obtained by removing reads with adapter, poly-N, or low-quality reads. High-quality clean reads were mapped onto the reference genome ASM465v1 using the TopHat2 tool.

### 4.3. Transcriptome Data Analysis

Raw reads of each sample were counted using the fastp tool for base quality, error rate, and A/T/G/C base content distribution. Clean reads were then obtained using SeqPrep (https://github.com/jstjohn/SeqPrep accessed on 13 January 2022) and Sickle (https://github.com/najoshi/sickle accessed on 13 January 2022) by removing reads with adapter, poly-N, or low-quality reads. At the same time, quality parameters of the clean data, including Q20, Q30, GC content, and sequence duplication level, were used for data filtering. All subsequent analyses were conducted using high-quality clean data. Clean reads were again evaluated using the fastp tool. High-quality clean reads were mapped onto the reference genome ASM465v1 using the TopHat2 tool. The overall quality of the comparison results was evaluated using RseQC-2.3.6, including sequencing saturation, sequencing coverage, distribution of reads in different regions, and distribution of reads in different chromosomes. The mapped reads were assembled into the transcriptome using StringTie software in a reference-based method. Novel transcripts were identified using the gffcompare software by comparing the transcriptome and reference genome. All genes/transcripts were annotated by mapping them to multiple databases, including NCBI protein nonredundant (NR), Swiss-Prot (https://www.uniprot.org accessed on 13 January 2022), Protein family (Pfam, http://pfam.xfam.org accessed on 13 January 2022), Clusters of Orthologous Groups of proteins (EggNOG, http://eggnog5.embl.de/#/app/home accessed on 13 January 2022), Gene Ontology (GO, Gene Ontology Resource), and Kyoto Encyclopedia of Genes and Genomes (KEGG, https://www.genome.jp/kegg accessed on 13 January 2022).

Gene expression levels were quantified using fragments per kilobases per million reads (FPKM) with the RSEM tool. Differential expression analysis was conducted using DESeq2, and thresholds of | log2(fold change) | ≤1 and *p* < 0.05 were used to assess significant differences. GO pathway enrichment analysis was performed using the Goatools, and R scripts were used for KEGG enrichment analysis; the groups with FDR ≤ 0.05 were considered significantly enriched. All raw data have been deposited in the Sequence Read Archive data repository (accession PRJNA787311) of the NCBI.

### 4.4. Expression Analysis by Quantitative Reverse-Transcription PCR

The total RNA of germinated seeds of SX628, SY01, and RHB under different treatments was extracted using an RNA extraction kit (TRIzol reagent, Takara, Kyoto, Japan). Total RNA (2 μg) from different samples was used for cDNA synthesis using TransScript^®^ One-Step gDNA Removal and cDNA Synthesis SuperMix (TransGen Biotech, Beijing, China). Real-time quantitative PCR (RT-qPCR) was performed using the SYBR^®^ Green Premix Pro Taq HS qPCR Kit (#AG11701, Accurate Biology, China) in the CFX Connect^TM^ Real-Time System (BIO-RAD, Hercules, CA, USA). Three biological replicates were used for each experiment. The relative expression levels of genes were calculated using the 2^−ΔΔCt^ method, and the *UBI* gene was used as an internal control. The primer sequences used for RT-qPCR are listed in Supplementary Table S8.

## Data Availability

All raw data have been deposited in the Sequence Read Archive data repository (accession PRJNA787311) of the NCBI.

## Supplementary Materials

The following supporting information can be downloaded at: https://www.mdpi.com/article/10.3390/ijms23031624/s1.
