# Supplementary material for: Molecular Insights into Salinity Responsiveness in Contrasting Genotypes of Rice at the Seedling Stage
Source: Int J Mol Sci. 2022 Jan 30;23(3):1624. doi: 10.3390/ijms23031624 (PMC8835730; doi:10.3390/ijms23031624)
Supplement: Supplementary file 1 [file ijms-23-01624-s001.zip › ijms-1542885-supplementary.pdf]

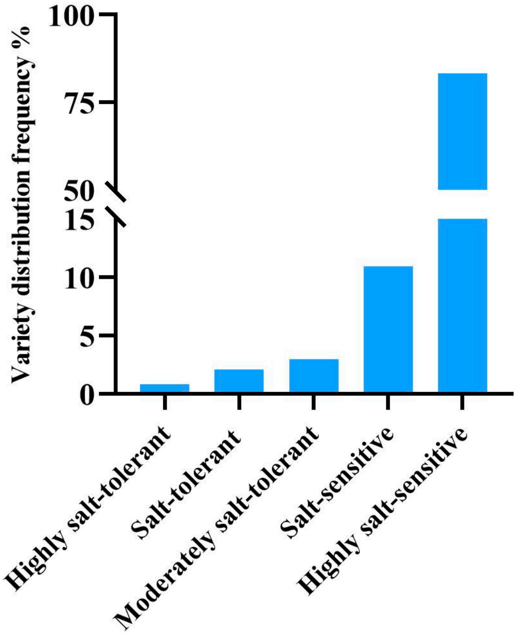

Figure S1 Percentage of different rice genotypes under salinity stress based on the relative damage rate parameter

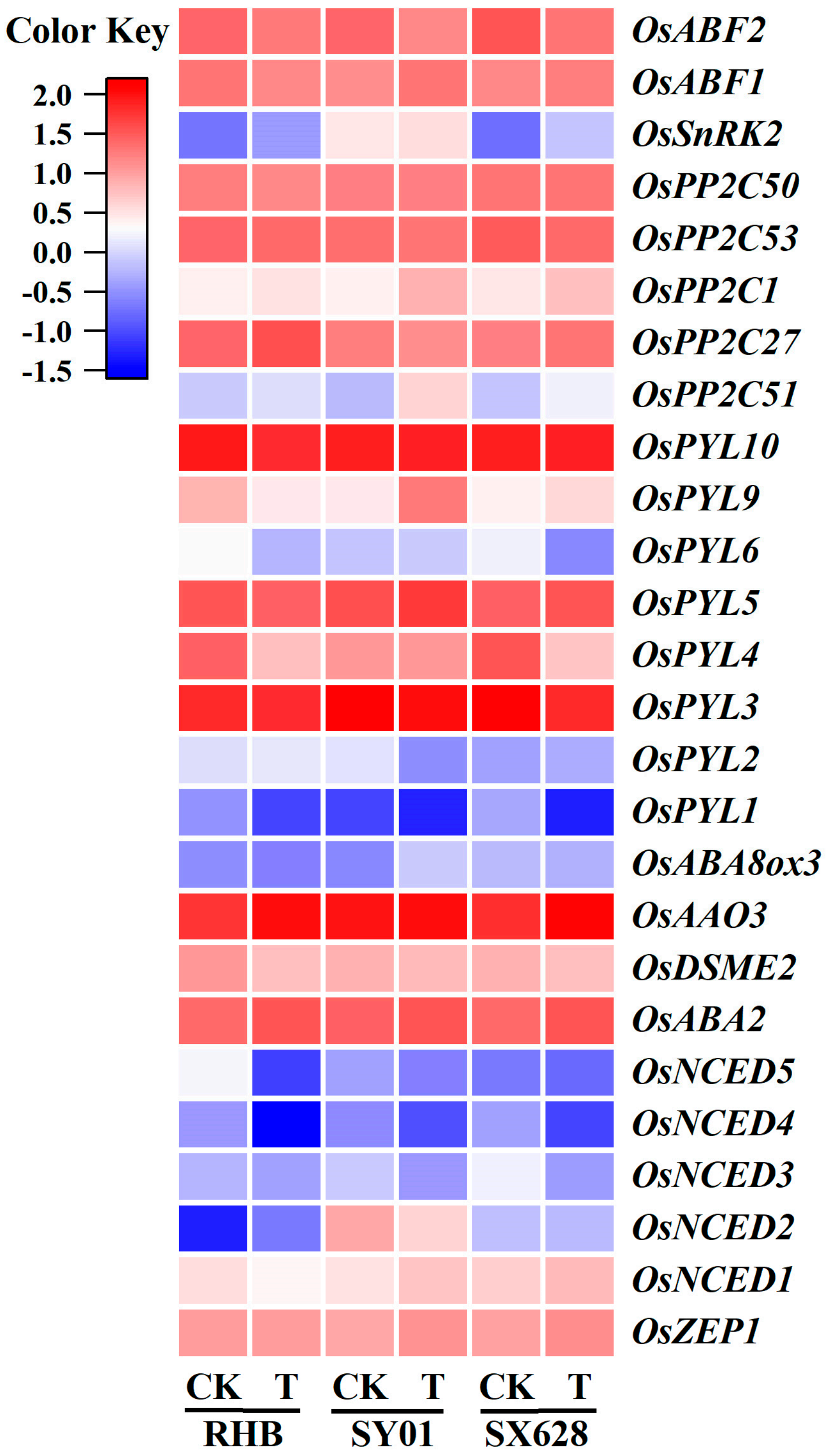

Figure S2 Heatmap of hormone-related genes differentially expressed under control and salinity stress

Table S1 Rankings of genotypes for their salt tolerance

NO.	Variety	Seedling rate-CK(%)	Seedling rate-150mM NaCl(%)	Relative damaging rate(%)	Salt tolerance
1	Sanxiang628	97.78	92.22	5.69	Highly salt-tolerant
2	IAPAR9(W27)	98	87.22	11	Highly salt-tolerant
3	R386	95	75	21.05	salt-tolerant
4	Guiyu12	100	78.9	21.1	salt-tolerant
5	Azucena	95.56	74.43	22.11	salt-tolerant
6	YL04	100	60	40	salt-tolerant
7	Nanfangheinuo	100	60	40	salt-tolerant
8	WD1790B	100	57.5	42.5	Moderately salt-tolerant
9	106B	97.78	55.56	43.18	Moderately salt-tolerant
10	ZZ-153	95	47.5	50	Moderately salt-tolerant
11	Meinianxian	97.50	45	53.85	Moderately salt-tolerant
12	Thailand xiaoxiangzhan	100	45.00	55	Moderately salt-tolerant
13	SY01	93.33	40	57.14	Moderately salt-tolerant
14	T165	95	40	57.89	Moderately salt-tolerant
15	Zhong4188(W3)	98.9	34.44	65.18	salt-sensitive
16	R778	97.50	32.50	66.67	salt-sensitive
17	Huahang31	100	32.5	67.5	salt-sensitive
18	WT17530A	95	30	68.42	salt-sensitive
19	WD18259A	92.5	27.5	70.27	salt-sensitive
20	Guangyouzhan	97.5	27.5	71.79	salt-sensitive
21	Xinhuangzhan	100	27.5	72.5	salt-sensitive
22	Hom Mali	100	27.5	72.5	salt-sensitive
23	Makan Youzhan	100	27.50	72.50	salt-sensitive
24	Batai Xiangzhan	100	27.5	72.5	salt-sensitive
25	Yuebiao5	95	25	73.68	salt-sensitive
26	C2	97.50	25	74.36	salt-sensitive
27	Yunlu108	97.5	25	74.36	salt-sensitive
28	ZZ-157	100	25	75	salt-sensitive
29	Zhongguangxiang 1	97.5	22.5	76.92	salt-sensitive
30	ZZ-176	97.50	22.50	76.92	salt-sensitive
31	Meixiangzhan 2	100	22.5	77.5	salt-sensitive
32	ZZ-93	100	22.50	77.50	salt-sensitive
33	R481	98.89	22.22	77.53	salt-sensitive
34	ZZ-26	80.00	17.50	78.13	salt-sensitive
35	Zhongkexilu5	95	20	78.95	salt-sensitive
36	WD18184A	95	20	78.95	salt-sensitive
37	Nanxiuruanzhan	95	20	78.95	salt-sensitive
38	RHB	98.9	20	79.8	salt-sensitive
39	Zaoxinzhan1	87.50	17.50	80.00	salt-sensitive
40	Wenxiangzhan	100	20	80	salt-sensitive

Table S2 Sample sequencing data quality summary in this study.

41	IR36	90	17.5	80.56	Highly salt-sensitive
42	WD18128A	67.5	12.5	81.48	Highly salt-sensitive
43	Minghui3301	97.50	17.50	82.05	Highly salt-sensitive
44	G525	100	17.5	82.5	Highly salt-sensitive
45	Guiyefeng	100	17.5	82.5	Highly salt-sensitive
46	zz-145	100	17.5	82.5	Highly salt-sensitive
47	ZH-01	90	15	83.33	Highly salt-sensitive
48	ZZ-118	95.00	15.00	84.21	Highly salt-sensitive
49	WT17533A	80	12.5	84.38	Highly salt-sensitive
50	ZZ-86	82.50	12.50	84.85	Highly salt-sensitive
51	WD18378A	97.78	14.44	85.23	Highly salt-sensitive
52	WT18913A	90	12.5	86.11	Highly salt-sensitive
53	ZZ-133	92.50	12.50	86.49	Highly salt-sensitive
54	ZZ-84	97.50	12.50	87.18	Highly salt-sensitive
55	Nongxiang32	100	12.5	87.5	Highly salt-sensitive
56	WT17407A	87.5	10	88.57	Highly salt-sensitive
57	WD18381A	92.5	10	89.2	Highly salt-sensitive
58	WD18158B	95	10	89.47	Highly salt-sensitive
59	WT18903A	97.5	10	89.74	Highly salt-sensitive
60	ZZ-32	97.50	10	89.74	Highly salt-sensitive
61	Nanjingzhan	100	10	90	Highly salt-sensitive
62	ZZ-69	97.50	7.50	92.31	Highly salt-sensitive
63	WD17106A	97.5	7.5	92.31	Highly salt-sensitive
64	GZ492	100	7.5	92.5	Highly salt-sensitive
65	WD17113A	100	7.5	92.5	Highly salt-sensitive
66	Wushanxiuzhan	100	7.5	92.5	Highly salt-sensitive
67	Zhenxiang	98.9	5.6	94.3	Highly salt-sensitive
68	B1(292B)	97.5	5	94.87	Highly salt-sensitive
69	R134	100	5	95	Highly salt-sensitive
70	Yuefengxinzhan	100	5	95	Highly salt-sensitive
71	D110	100	5	95.00	Highly salt-sensitive
72	D188	100	5	95	Highly salt-sensitive
73	Xinxiangzhan	100	5	95	Highly salt-sensitive
74	ZZ-182	100	5	95.00	Highly salt-sensitive
75	ZZ-35	100	5	95.00	Highly salt-sensitive
76	Yueyousimiao	100	5	95	Highly salt-sensitive
77	ZZ-53	100	5	95.00	Highly salt-sensitive
78	R1173	100	5	95	Highly salt-sensitive
79	D94	100	3.33	96.67	Highly salt-sensitive
80	Xiangya	82.5	2.5	96.97	Highly salt-sensitive
81	D143	90	2.5	97.22	Highly salt-sensitive
82	ZZ-19	97.50	2.50	97.44	Highly salt-sensitive
83	ZZ-74	97.50	2.50	97.44	Highly salt-sensitive
84	WD18382A	97.5	2.5	97.44	Highly salt-sensitive

85	Yuete13	100	2.5	97.5	Highly salt-sensitive
86	Minghui63	100	2.5	97.5	Highly salt-sensitive
87	ZH-02	100	2.5	97.5	Highly salt-sensitive
88	B176	100	2.50	97.50	Highly salt-sensitive
89	DUS baohuhang	100	2.5	97.5	Highly salt-sensitive
90	ZZ-273	100	2.50	97.50	Highly salt-sensitive
91	Yuxiangyouzhan	100	2.5	97.5	Highly salt-sensitive
92	WD1791B	97.78	2.22	97.73	Highly salt-sensitive
93	Chuanqi	94.44	0	100	Highly salt-sensitive
94	R900	95	0	100	Highly salt-sensitive
95	9311	100	0	100	Highly salt-sensitive
96	Hua15	98.9	0	100	Highly salt-sensitive
97	Huazhang	87.5	0	100	Highly salt-sensitive
98	RH3B	92.5	0	100	Highly salt-sensitive
99	R135	100	0	100	Highly salt-sensitive
100	Youhui168	100	0	100	Highly salt-sensitive
101	Youhui131	98.33	0	100	Highly salt-sensitive
102	Zhongkexilu4	100	0	100	Highly salt-sensitive
103	Lijiang xintuan heigu	65	0	100	Highly salt-sensitive
104	Digu	100	0	100	Highly salt-sensitive
105	TeTep(W1)	100	0	100	Highly salt-sensitive
106	Amol3(W2)	100	0	100	Highly salt-sensitive
107	TaifengB	77.5	0	100	Highly salt-sensitive
108	Zihui100(W5)	95	0	100	Highly salt-sensitive
109	Katy(W6)	93.33	0	100	Highly salt-sensitive
110	IR64(W8)	100	0	100	Highly salt-sensitive
111	NanyangZhan(W10)	75	0	100	Highly salt-sensitive
112	Basmati370(W11)	95	0	100	Highly salt-sensitive
113	IR58025B(W12)	97.5	0	100	Highly salt-sensitive
114	Jiangxisimiao(W13)	97.50	0	100	Highly salt-sensitive
115	T356	100	0	100	Highly salt-sensitive
116	Chenglongshuijingmi(W20)	85	0	100	Highly salt-sensitive
117	Lemont(W23)	92.5	0	100	Highly salt-sensitive
118	Guang8B	100	0	100	Highly salt-sensitive
119	Huajingxian74	100	0	100	Highly salt-sensitive
120	20151284C	80	0	100	Highly salt-sensitive
121	G510	90	0	100	Highly salt-sensitive
122	YexiangB	97.5	0	100	Highly salt-sensitive
123	G569	73.33	0	100	Highly salt-sensitive
124	GZ484	100	0	100	Highly salt-sensitive
125	GZ493	87.5	0	100	Highly salt-sensitive
126	B2	97.5	0	100	Highly salt-sensitive
127	WD17117A	95	0	100	Highly salt-sensitive
128	WD17118A	100	0	100	Highly salt-sensitive

129	WD17120A	100	0	100	Highly salt-sensitive
130	WD17124A	97.5	0	100	Highly salt-sensitive
131	WD18201A	96.67	0	100	Highly salt-sensitive
132	WD18299A	100	0	100	Highly salt-sensitive
133	WD18313A	100	0	100	Highly salt-sensitive
134	WD18334A	97.5	0	100	Highly salt-sensitive
135	WD18339A	95	0	100	Highly salt-sensitive
136	WD18355A	100	0	100	Highly salt-sensitive
137	WD18360A	100	0	100	Highly salt-sensitive
138	WD18423A	95	0	100	Highly salt-sensitive
139	WD18450A	97.5	0	100	Highly salt-sensitive
140	WD184538A	85.00	0	100	Highly salt-sensitive
141	WD18466A	88.33	0	100	Highly salt-sensitive
142	WD1868A	95.00	0	100	Highly salt-sensitive
143	WD18277A	92.50	0	100	Highly salt-sensitive
144	WT17250A	87.5	0	100	Highly salt-sensitive
145	WT17397A	100	0	100	Highly salt-sensitive
146	Huanghuazhan	95.00	0	100	Highly salt-sensitive
147	WT17538A	90	0	100	Highly salt-sensitive
148	WT17545A	95	0	100	Highly salt-sensitive
149	WD18153A	90	0	100	Highly salt-sensitive
150	Jingnongsimiao	100	0	100	Highly salt-sensitive
151	Cungunuo	95	0	100	Highly salt-sensitive
152	Kemainuo	92.50	0	100	Highly salt-sensitive
153	Huangguangyouzhan	97.5	0	100	Highly salt-sensitive
154	Lunhui01	95	0	100	Highly salt-sensitive
155	Aixiuzhan	90	0	100	Highly salt-sensitive
156	Zhongjuanxian	97.50	0	100	Highly salt-sensitive
157	Zhengchengsimiao	95	0	100	Highly salt-sensitive
158	Zhudao	90	0	100	Highly salt-sensitive
159	Qiyezhan	82.5	0	100	Highly salt-sensitive
160	B111	100	0	100	Highly salt-sensitive
161	Hefengsimiao	100	0	100	Highly salt-sensitive
162	Huanglisimiao	97.5	0	100	Highly salt-sensitive
163	Huangsizhan	90	0	100	Highly salt-sensitive
164	Huangxiuzhan	92.5	0	100	Highly salt-sensitive
165	Huangyinzhan	100	0	100	Highly salt-sensitive
166	lvyuanzhan	100	0	100	Highly salt-sensitive
167	Nanguizhan	100	0	100	Highly salt-sensitive
168	B174	100	0	100	Highly salt-sensitive
169	Nanyouzhan	100	0	100	Highly salt-sensitive
170	Wushansimiao	97.50	0	100	Highly salt-sensitive
171	Yuehesimiao	95	0	100	Highly salt-sensitive
172	Yuehuasimiao	97.5	0	100	Highly salt-sensitive

173	Huangruanzhan	97.5	0	100	Highly salt-sensitive
174	Yuenongsimiao	92.5	0	100	Highly salt-sensitive
175	D25	100	0	100	Highly salt-sensitive
176	D53	97.5	0	100	Highly salt-sensitive
177	D130	99	0	100	Highly salt-sensitive
178	D140	97.78	0	100	Highly salt-sensitive
179	D141	65.00	0	100	Highly salt-sensitive
180	D142	95	0	100	Highly salt-sensitive
181	D180	100	0	100	Highly salt-sensitive
182	D191	100	0	100	Highly salt-sensitive
183	D194	95	0	100	Highly salt-sensitive
184	D213	92.5	0	100	Highly salt-sensitive
185	D215	97.5	0	100	Highly salt-sensitive
186	D216	82.50	0	100	Highly salt-sensitive
187	P79	92.50	0	100	Highly salt-sensitive
188	D221	80.00	0	100	Highly salt-sensitive
189	D231	100	0	100	Highly salt-sensitive
190	D279	100	0	100	Highly salt-sensitive
191	D289	96.67	0	100	Highly salt-sensitive
192	D301	100	0	100	Highly salt-sensitive
193	D302	100	0	100	Highly salt-sensitive
194	Taiguoxiangmi	100	0	100	Highly salt-sensitive
195	Guixiang3	90	0	100	Highly salt-sensitive
196	Guifengxiangzhan	100	0	100	Highly salt-sensitive
197	ZMZ-42	95	0	100	Highly salt-sensitive
198	ZMZ-43	97.5	0	100	Highly salt-sensitive
199	19xiang	100	0	100	Highly salt-sensitive
200	ZZ-105	95	0	100	Highly salt-sensitive
201	ZZ-106	98	0	100	Highly salt-sensitive
202	ZZ-108	98	0	100	Highly salt-sensitive
203	ZZ-110	100	0	100	Highly salt-sensitive
204	Gufengzhan	95	0	100	Highly salt-sensitive
205	ZZ-113	100	0	100	Highly salt-sensitive
206	ZZ-121	97.50	0	100	Highly salt-sensitive
207	ZZ-126	95.00	0	100	Highly salt-sensitive
208	ZZ-128	92.50	0	100	Highly salt-sensitive
209	ZZ-129	97.50	0	100	Highly salt-sensitive
210	ZZ-139	100	0	100	Highly salt-sensitive
211	ZZ-141	100	0	100	Highly salt-sensitive
212	ZZ-146	100	0	100	Highly salt-sensitive
213	ZZ-164	100	0	100	Highly salt-sensitive
214	ZZ-17	52.50	0	100	Highly salt-sensitive
215	ZZ-172	100	0	100	Highly salt-sensitive
216	ZZ-177	97.50	0	100	Highly salt-sensitive

217	ZZ-181	100	0	100	Highly salt-sensitive
218	ZZ-184	92.50	0	100	Highly salt-sensitive
219	ZZ-185	100	0	100	Highly salt-sensitive
220	ZZ-24	95.00	0	100	Highly salt-sensitive
221	ZZ-27	85.00	0	100	Highly salt-sensitive
222	ZZ-36	97.50	0	100	Highly salt-sensitive
223	ZZ-37	95	0	100	Highly salt-sensitive
224	ZZ-38	100	0	100	Highly salt-sensitive
225	ZZ-46	97.50	0	100	Highly salt-sensitive
226	Dingfeng	97.5	0	100	Highly salt-sensitive
227	Huahang48	87.5	0	100	Highly salt-sensitive
228	Liu Chang	87.5	0	100	Highly salt-sensitive
229	Guiyefeng	97.5	0	100	Highly salt-sensitive
230	ZZ-61	97.50	0	100	Highly salt-sensitive
231	R1179	100	0	100	Highly salt-sensitive
232	20S-ZZ-64	92.50	0	100	Highly salt-sensitive
233	20S-ZZ-66	97.50	0	100	Highly salt-sensitive
234	20S-ZZ-77	100	0	100	Highly salt-sensitive
235	20S-ZZ-79	95	0	100	Highly salt-sensitive
236	20S-ZZ-8	95	0	100	Highly salt-sensitive
237	20S-ZZ-85	100	0	100	Highly salt-sensitive
238	20S-ZZ-91	97.50	0	100	Highly salt-sensitive

Sample	Raw reads	Raw bases	Clean reads	Clean bases	Error rate	Q20	Q30	GC content
RHB_CK1	56974394	8603133494	56128488	8301738277	0.0261	97.49	93.25	51.62
RHB_CK2	42966458	6487935158	42521442	6311929069	0.0249	98.06	94.28	50.1
RHB_CK3	48940690	7390044190	48373142	7175727170	0.0256	97.77	93.65	50.89
RHB_T1	54679726	8256638626	53784736	7908648545	0.0262	97.41	93.13	51.17
RHB_T2	53035220	8008318220	52339176	7551761632	0.0254	97.78	93.89	51.85
RHB_T3	58291492	8802015292	57498198	8458084998	0.0256	97.65	93.66	51.19
SY01_CK1	54214254	8186352354	53480298	7893063446	0.026	97.54	93.34	51.6
SY01_CK2	58874912	8890111712	58194586	8611580894	0.0256	97.7	93.71	51.37
SY01_CK3	58866948	8888909148	58220644	8600466378	0.0254	97.8	93.89	51.77
SY01_T1	46886092	7079799892	46179696	6826531691	0.0259	97.54	93.42	51.61
SY01_T2	51731316	7811428716	51018206	7498799674	0.0261	97.48	93.19	51.87
SY01_T3	48141076	7269302476	47507460	7013608887	0.0259	97.58	93.45	52.01
SX628_CK1	50485078	7623246778	49723826	7274294900	0.0262	97.45	93.13	50.16
SX628_CK2	52274096	7893388496	51493102	7528146937	0.0256	97.69	93.66	50.43
SX628_CK3	50350176	7602876576	49578014	7283278497	0.0264	97.37	92.95	50.7
SX628_T1	52870194	7983399294	52242570	7726800481	0.0256	97.67	93.63	52.16
SX628_T2	61031390	9215739890	60285742	8934331645	0.0258	97.6	93.48	51.22
SX628_T3	47020366	7100075266	46354740	6835070963	0.0262	97.44	93.1	51.25

Table S3 Sample sequencing data mapped summary in this study.

Sample	Total reads	Total mapped	Multiple mapped	Uniquely mapped
RHB_CK1	56128488	50383540(89.76%)	7978528(14.21%)	42405012(75.55%)
RHB_CK2	42521442	38319757(90.12%)	2480796(5.83%)	35838961(84.28%)
RHB_CK3	48373142	43349977(89.62%)	2176451(4.5%)	41173526(85.12%)
RHB_T1	53784736	49036699(91.17%)	4147016(7.71%)	44889683(83.46%)
RHB_T2	52339176	47711363(91.16%)	5178925(9.89%)	42532438(81.26%)
RHB_T3	57498198	52482079(91.28%)	3374772(5.87%)	49107307(85.41%)
SY01_CK1	53480298	49124550(91.86%)	3744720(7.0%)	45379830(84.85%)
SY01_CK2	58194586	53230492(91.47%)	3071089(5.28%)	50159403(86.19%)
SY01_CK3	58220644	53561783(92.0%)	3036681(5.22%)	50525102(86.78%)
SY01_T1	46179696	42515390(92.07%)	2931144(6.35%)	39584246(85.72%)
SY01_T2	51018206	47257628(92.63%)	3534745(6.93%)	43722883(85.7%)
SY01_T3	47507460	43609538(91.8%)	3162018(6.66%)	40447520(85.14%)
SX628_CK1	49723826	45545296(91.6%)	2677462(5.38%)	42867834(86.21%)
SX628_CK2	51493102	47603877(92.45%)	4508497(8.76%)	43095380(83.69%)
SX628_CK3	49578014	45471336(91.72%)	2853518(5.76%)	42617818(85.96%)
SX628_T1	52242570	48384171(92.61%)	2665274(5.1%)	45718897(87.51%)
SX628_T2	60285742	55926843(92.77%)	3444604(5.71%)	52482239(87.06%)
SX628_T3	46354740	42997470(92.76%)	3005952(6.48%)	39991518(86.27%)

Table S4 Different type of transcripts with class code were detected using Gffcompare

**Class_code**	**Description**	**Number**
=	Complete match of intron chain	42029
i	Transfrag falling entirely within a reference intron	212
j	Potentially novel isoform (fragment):at least one splice junction is shared with a reference transcript	25065
o	Generic exonic overlap with a referfence transcript	3672
u	Unknown, intergenic transcript	5443
x	Exonic overlap with reference on the opposite strand	1911

Table S5 Alternative splicing(AS) events in the transcriptome of different samples

**Sample name**	**SE**	**RI**	**A5SS**	**A3SS**	**MXE**	**Total**
RHB_CK1	11628	1639	2093	3714	800	19874

RHB_CK2	11416	1564	2017	3534	760	19291
RHB_CK3	11669	1652	2112	3707	817	19957
RHB_T1	12615	1932	2324	3963	989	21823
RHB_T2	12558	1873	2285	3921	968	21605
RHB_T3	12612	1958	2320	3957	987	21834
SY01_CK1	11730	1987	2297	3939	895	20848
SY01_CK2	11873	2015	2307	3985	890	21070
SY01_CK3	11731	1975	2295	3936	892	20829
SY01_T1	12746	2053	2389	4014	1027	22229
SY01_T2	12748	2082	2378	4023	1041	22272
SY01_T3	12802	2084	2391	4029	1023	22329
SX628_CK1	12157	2101	2399	4054	947	21658
SX628_CK2	12102	2018	2355	3976	936	21387
SX628_CK3	12149	2056	2389	4030	940	21564
SX628_T1	13016	2141	2437	4075	1088	22757
SX628_T2	13013	2162	2457	4085	1083	22800
SX628_T3	13075	2135	2454	4091	1099	22854

SE: Skipped exon; RI: Retained intron; A5SS: Alternative 5’ splice site; A3SS: Alternative 3’ splice site; MXE: Mutually exclusive exons.

Table S6 19 upregulated genes were identified in starch and sucrose metabolism pathway under control and salinity treatments

Accession Number	Gene Name	RHB CK vs T		SY01 CK vs T		SX628 CK vs T		Description
		Log2FC	Padjust	Log2FC	Padjust	Log2FC	Padjust	
Os01g0190400	*OsHXK8*	1.36	1.05E-05	1.23	0.000461	2.14	0.000114	Phosphotransferase
Os05g0518600	*OsTPS1*	1.01	1.11E-07	1.50	8.22E-06	1.51	2.15E-15	Trehalose-6-phosphate synthase
Os04g0513100	*OsBGLU14*	3.86	2.29E-09	3.68	2.69E-07	4.33	1.78E-12	Monolignol beta-glucoside homologue without catalytic acid/base
Os03g0340500	*OsSUS4*	1.46	1.85E-08	1.22	4.04E-05	2.94	1.9E-89	Sucrose synthase
Os05g0580000	*OsAGPL1*	1.65	3.59E-08	2.38	4.06E-25	2.82	1.45E-12	Glucose-1-phosphate adenylyltransferase
Os09g0298200	*OsAGPS1*	1.56	3.39E-10	1.43	2.49E-15	2.59	5.37E-33	Glucose-1-phosphate adenylyltransferase
Os06g0160700	*OsSSI*	2.28	1.04E-17	1.75	7.46E-19	2.69	2.96E-20	Soluble starch synthase 1, chloroplastic/amyloplastic
Os06g0229800	*OsSSIIa*	2.86	4.58E-07	4.27	2.8E-16	4.08	7.48E-18	Starch synthase, chloroplastic/amyloplastic
Os07g0412100	*OsGBSSII;*	1.64	2.83E-20	2.00	1.22E-25	1.95	1.8E-30	Starch synthase, chloroplastic/amyloplastic
Os01g0851700	*OsPho2*	2.36	9.53E-16	1.97	1.73E-18	3.40	1.43E-42	Alpha-1,4 glucan phosphorylase
Os03g0758100	*OsPho1*	2.73	4.65E-31	3.01	1.63E-61	4.11	9.64E-36	Alpha-1,4 glucan phosphorylase
Os07g0627000	*OsDPE1*	1.65	2.7E-16	1.45	8.22E-09	1.69	1.33E-12	4-alpha-glucanotransferase
Os02g0528200	*OsBEIIb*	1.81	3.57E-06	1.74	1.53E-13	3.53	3.56E-15	Starch branching enzyme 3
Os04g0409200	*OsBEIIa*	2.35	2.37E-26	2.17	2.78E-29	3.29	3.85E-26	1,4-alpha-glucan-branching enzyme 2
Os04g0554000	*OsTPP5*	1.77	0.005428	3.12	1.74E-07	1.69	0.03599	Trehalose 6-phosphate phosphatase
Os09g0469400	*OsISA3;*	2.15	3.1E-12	2.26	6.16E-22	2.21	1.44E-23	Glycogen operon protein glgX
Os05g0393700	*OsISA2*	1.72	3.56E-08	1.08	0.000406	2.78	3.69E-48	Alpha amylase, catalytic domain containing protein
Os08g0520900	*OsISA1*	2.03	1.86E-08	1.60	4.99E-17	3.10	1.46E-37	Alpha amylase, catalytic domain containing protein
Os01g0357400	*Osamy-c*	1.32	0.009016	2.73	9.05E-57	3.75	0.007756	Alpha-amylase isozyme C

Table S7 Hormone-related genes differentially expressed under control and salinity stress

Accession Number	Gene Name	RHB CK vs T		SY01 CK vs T		SX628 CK vs T		Description
		Log2FC	Padjust	Log2FC	Padjust	Log2FC	Padjust	
Os04g0448900	*ZEP1*	0.26	0.375741	0.45	0.014422	0.69	0.002159	Zeaxanthin epoxidase, chloroplastic
Os02g0704000	*NCED1*	-0.51	0.419290	0.61	0.279626	0.89	0.094703	9-cis-epoxycarotenoid dioxygenase
Os12g0435200	*NCED2*	2.49	0.214692	-1.19	0.006452	0.06	0.977790	9-cis-epoxycarotenoid dioxygenase
Os03g0645900	*NCED3*	-0.25	0.820077	-1.27	0.197055	-1.71	0.019469	9-cis-epoxycarotenoid dioxygenase
Os07g0154100	*NCED4*	-3.54	0.086338	-1.53	0.381584	-1.98	0.345497	9-cis-epoxycarotenoid dioxygenase
Os12g0617400	*NCED5*	-4.11	0.005574	-0.83	0.664234	-0.09	0.956351	9-cis-epoxycarotenoid dioxygenase
Os03g0810800	*ABA2*	0.65	0.023344	0.22	0.299843	0.73	0.000355	Xanthoxin dehydrogenase
Os03g0125100	*DSME2*	-0.74	0.104410	-0.27	0.553333	-0.16	0.667089	β-Carotene Hydroxylase
Os03g0790900	*AAO3*	1.10	0.005164	0.19	0.450732	1.27	0.000591	aldehyde oxidase
Os09g0457100	*ABA8ox3*	-0.03	0.987742	1.71	0.055630	0.02	0.986591	Abscisic acid 8'-hydroxylase 3
Os01g0827800	*PYL1*	-1.65	1.000000	-0.66	1.000000	-2.78	1.000000	ABA receptor 8
Os02g0226801	*PYL2*	0.45	0.790862	-1.94	0.273728	0.50	0.802816	ABA receptor 10
Os02g0255500	*PYL3*	-0.03	0.951982	-0.32	0.186817	-0.87	0.006403	ABA receptor 2
Os03g0297600	*PYL4*	-1.95	0.000073	-0.04	0.948409	-2.43	0.000000	ABA receptor 6
Os05g0213500	*PYL5*	-0.17	0.525032	0.33	0.128173	0.19	0.391163	rice orthologue of the ABA receptor
Os05g0473000	*PYL6*	-1.59	0.197725	0.11	0.959968	-2.34	0.104892	pyrabactin resistance-like abscisic acid receptor
Os06g0562200	*PYL9*	-1.14	0.111957	2.82	0.000000	0.92	0.351498	ABA receptor 11
Os10g0573400	*PYL10*	-0.12	0.586230	-0.16	0.530212	0.14	0.633604	ABA receptor 9

Os05g0572700	*PP2C51*	0.79	0.412249	2.74	0.000000	1.32	0.062696	protein phosphatase 2C clade A protein
Os02g0799000	*PP2C27*	0.82	0.157120	-0.27	0.387424	0.60	0.002655	type 2C protein phosphatase
Os09g0325700	*PP2C1*	0.56	0.236117	1.61	0.055304	1.07	0.021210	Protein Phosphatase 2C
Os05g0592800	*PP2C53*	-0.09	0.734360	-0.16	0.450205	-0.17	0.408179	clade A type 2C protein phosphatase
Os05g0537400	*PP2C50*	0.03	0.913579	0.12	0.827967	0.08	0.762883	clade A type 2C protein phosphatase
Os04g0629300	*SnRK2*	0.90	0.410880	0.17	0.597341	2.49	0.000309	Snf2 family protein
Os01g0867300	*ABF1*	-0.19	0.517333	0.73	0.013389	0.56	0.000989	bZIP transcription factor, Abiotic stress response, ABA signaling, Suppressor of floral transition upon drought stres
Os02g0766700	*ABF2*	-0.32	0.118147	-0.95	0.023628	-0.63	0.005625	bZIP transcription factor, Regulation of ABA signaling and biosynthesis, Drought resistanc

Table S8 List of Primers used in this study

Application	Primer	Sequence（5'-3'）
qRT-PCR	OsSUS4-F	ATGTAAACAAGACCCCAACCA
qRT-PCR	OsSUS4-R	CAGTGTGCCTCATCTACTGC
qRT-PCR	OsAGPL1-F	CTTGCGTGAATGTACCATCG
qRT-PCR	OsAGPL1-R	TACACCAATGGGGACCTTGC
qRT-PCR	OsBEIIb-F	ATTTGGCAGGATCCATCACAC
qRT-PCR	OsBEIIb-R	AGCATAGACAACGCAGGTTC
qRT-PCR	OsHAK1-F	GGCCCAACTCGTCCATCC
qRT-PCR	OsHAK1-R	ACGTGATCCCAACCTTGAGC
qRT-PCR	OsHAK19-F	CAACGCAGCCAAGATAGCAC
qRT-PCR	OsHAK19-R	GCATCGAGAACAGCACGAAC
qRT-PCR	OsHAK5-F	AGACCAACACACGACGGAAC
qRT-PCR	OsHAK5-R	ACGTCATTCCTACCCGCAAC
qRT-PCR	OsHAK22-F	ACATACCGGTGGCCATGTCG
qRT-PCR	OsHAK22-R	GCTCCCTCCGAGACACAAGC
